# The effect of forearm posture on wrist flexion in computer workers with chronic upper extremity musculoskeletal disorders

**DOI:** 10.1186/1471-2474-9-47

**Published:** 2008-04-11

**Authors:** Ronald A Burgess, R Terry Thompson, Gary B Rollman

**Affiliations:** 1 Department of Medical Biophysics, University of Western Ontario, London, Ontario, Canada; 2 Imaging Division, Lawson Health Research Institute, London, Ontario, Canada; 3 Department of Psychology, University of Western Ontario

## Abstract

**Background:**

Occupational computer use has been associated with upper extremity musculoskeletal disorders (UEMSDs), but the etiology and pathophysiology of some of these disorders are poorly understood. Various theories attribute the symptoms to biomechanical and/or psychosocial stressors. The results of several clinical studies suggest that elevated antagonist muscle tension may be a biomechanical stress factor. Affected computer users often exhibit limited wrist range of motion, particularly wrist flexion, which has been attributed to increased extensor muscle tension, rather than to pain symptoms. Recreational or domestic activities requiring extremes of wrist flexion may produce injurious stress on the wrist joint and muscles, the symptoms of which are then exacerbated by computer use. As these activities may involve a variety of forearm postures, we examined whether changes in forearm posture have an effect on pain reports during wrist flexion, or whether pain would have a limiting effect on flexion angle.

**Methods:**

We measured maximum active wrist flexion using a goniometer with the forearm supported in the prone, neutral, and supine postures. Data was obtained from 5 subjects with UEMSDs attributed to computer use and from 13 control subjects.

**Results:**

The UEMSD group exhibited significantly restricted wrist flexion compared to the control group in both wrists at all forearm postures with the exception of the non-dominant wrist with the forearm prone. In both groups, maximum active wrist flexion decreased at the supine forearm posture compared to the prone posture. No UEMSD subjects reported an increase in pain symptoms during testing.

**Conclusion:**

The UEMSD group exhibited reduced wrist flexion compared to controls that did not appear to be pain related. A supine forearm posture reduced wrist flexion in both groups, but the reduction was approximately 100% greater in the UEMSD group. The effect of a supine forearm posture on wrist flexion is consistent with known biomechanical changes in the distal extensor carpi ulnaris tendon that occur with forearm supination. We infer from these results that wrist extensor muscle passive tension may be elevated in UEMSD subjects compared to controls, particularly in the extensor carpi ulnaris muscle. Measuring wrist flexion at the supine forearm posture may highlight flexion restrictions that are not otherwise apparent.

## Background

Occupational hand use has been associated with chronic upper limb pain and dysfunction, but the issue of causality remains controversial [1]. However, recent reviews of the epidemiological literature have concluded that there is a moderate, but consistent association between computer use and hand/arm symptoms [2,3]. Some symptoms may be consistent with specific clinical diagnoses (e.g., carpal tunnel syndrome (CTS), lateral epicondylitis), but frequently the symptoms are non-specific (e.g., myalgia) [4]. A prospective study of computer users found that 50% of hand/arm symptoms were non-specific [5]. This collection of specific and non-specific symptoms is known by a variety of labels including repetitive strain injury (RSI), cumulative trauma disorder (CTD), or work-related upper extremity disorder (WRUED). These labels are controversial, as they either imply causation or signify the presence of a disorder for which objective signs of pathology are often lacking. While we appreciate that no single term adequately encompasses the various specific and non-specific upper limb symptoms encountered in clinical practice [6], in this paper we use upper extremity musculoskeletal disorder (UEMSD) only to categorize the symptomatic subjects enrolled in our study. Current hypotheses ascribe the symptoms of UEMSD to muscle/tendon/soft-tissue damage, neurogenic disorders, or psychogenic causes [7,8], presumed to arise from biomechanical or psychosocial stressors [1].

The proliferation of computers in the workplace has coincided with an increase in the number of people reporting UEMSD attributed to computer use. Estimates of the prevalence of computer-related UEMSD vary depending on the diagnostic criteria used [9-11], but the fraction of computer workers who develop severe or disabling symptoms does not appear to be large. This implies differences in individual susceptibility or the influence of other etiological agents. There can be little doubt that computer use exacerbates upper limb symptoms, but whether these symptoms arise as a consequence of an unidentifiable injury sustained in the workplace or elsewhere is unknown. Hadler [12] has proposed that the wrist is susceptible only to "violence from without" and violence from "performance at the extremes of tissue tolerance". However, there exists a third possibility: that the wrist is susceptible to violence (or stress) from *within*. Individuals with UEMSD who engage in repetitive work (computer users, factory workers and instrumental musicians) often have limited wrist range of motion (ROM), which has been attributed to increased antagonist muscle tension [13-17]. Increased antagonist muscle tension in the upper limb may be a source of biomechanical stress during both occupational and non-occupational activities.

In the previous studies, increased antagonist muscle tension was inferred from limitations in wrist ROM [13-17]. Pain is known to have a limiting effect on joint mobility, but pain elicited during testing was mentioned in only one study [14]. There seems to be general agreement by the various authors that wrist ROM was limited by increased antagonist muscle tension [13,14,16,17] or ligament/muscle/tendon shortening [15] although there is no consensus on whether this represents an increase in active or passive tension. There is some evidence for increased agonist muscle activity in upper extremity disorders [18,19] as well as a positive relationship between antagonist muscle activity and trait anxiety [20], but we are not aware of any studies demonstrating increased antagonist muscle activity in UEMSD patients. It has also been suggested that the increase in antagonist muscle passive tension resulted from replacement of Type II muscle fibers with shorter Type I fibers in response to chronic muscle activity [13]. However, the results of studies relating changes in muscle fiber-type distribution to work-related myalgia have been contradictory [21-23].

Despite the uncertainty regarding the underlying mechanism, an increase in antagonist muscle tension would clearly affect wrist joint dynamics. Wrist flexion in affected computer users is often impaired compared to normative values or to the unaffected wrist [13,16,17]. Increased wrist extensor muscle tension would demand increased flexor muscle activity during wrist flexion, thereby increasing muscle and wrist joint loading. This increased loading may produce sufficient stress to elicit symptoms in the joint and muscle, particularly at the extremes of wrist flexion. Although computer use involves primarily wrist extension rather than flexion, other domestic or recreational activities that require repetitive or forceful wrist flexion may strain the joint or muscles, the symptoms of which are exacerbated by and then attributed to occupational computer use.

Wrist ROM is normally measured with the forearm prone and the elbow flexed 90 degrees [24], which is the posture normally assumed during computer use. Several of the previous studies did not specify the forearm postures used during flexion testing, particularly the study noting an increase in pain reported during testing [14]. Recreational or domestic activities involving a variety of forearm postures with the elbow fully extended may affect joint loading and symptoms. Therefore, it would be useful to quantify wrist flexion at several forearm postures with the elbow extended. Techniques for measuring joint ROM may be passive – using examiner applied force, or active – using subject generated force. The previous studies of wrist ROM [13-17] used either one or both techniques, and passive flexion was always greater than active flexion. We used active measures of wrist ROM to minimize any potential discomfort to the subjects. The objective of this study was to determine whether changes in forearm posture would affect pain reports during maximum active wrist flexion, or whether pain would have a limiting effect on flexion angle.

## Methods

### Subjects

5 subjects (4 female, 1 male, mean age = 43, SD = 5.1 yrs.) who reported chronic (> 1 year duration) UEMSDs that they attributed to occupational computer use were recruited from a local RSI support group and the community. Two subjects reported bilateral symptoms, and three reported unilateral dominant-side symptoms. All subjects reported forearm muscle pain, and all but one reported wrist pain and/or pain in the hand or fingers. These symptoms are consistent with UEMSD, and the subjects had received a variety of diagnoses (RSI, tendonosis, CTS) by either their family physician or a specialist. No UEMSD subjects reported any previous trauma, and all were currently working except for one female subject who was off work due to symptoms. The control group consisted of thirteen subjects (7 female, 6 male, mean age = 46, SD = 17 yrs.) with no history of wrist or forearm symptoms. Only two of the control subjects used computers extensively in their occupations. All subjects were right-hand dominant except for one male control subject. Our Institutional Ethics Review Board approved the study, and informed consent was obtained from each subject.

### Materials & Procedure

A custom wrist support was used to maintain the elbow in full extension, the wrist in neutral radioulnar deviation, and the forearm prone, neutral, or supine. The support was designed to contact only the wrist and elbow to prevent compression of the forearm musculature. Neutral radioulnar deviation was maintained with an adjustable guide bar during testing with the forearm supine, by contact with the hand support with the forearm neutral, and visually with the forearm prone. The wrist support's height was adjusted to align the forearm parallel to the floor at shoulder height with the subject seated. A 6" universal goniometer (Medelco model DIA 503) was used to determine maximum active wrist flexion as the angle formed between the midline of the long axis of the radius and the dorsal aspect of the 3rd metacarpal. This deviation from the normal testing procedure [24], where the dorsal aspect of the radius anchors one arm of the goniometer, was necessary as the wrist support interfered with this positioning when the forearm was supine. This technique has been previously shown to be reliable for measures of wrist extension [25]. The subjects were instructed to relax their fingers and flex only their wrists as far as possible while maintaining neutral radioulnar deviation, and to report any symptoms that arose or were exacerbated during the maneuver. The sequence was randomized with respect to the initial wrist measured and forearm posture. Maximum active wrist flexion was measured once at each of the three forearm postures. The wrist flexion data were analyzed with a 2 × 2 × 3 (Group × Side × Posture) mixed analysis of variance using SPSS version 16, (SPPS Inc., Chicago, IL) with side and posture as the repeated measures. Any significant effects (two-tailed) were followed by a series of *post hoc *means comparisons adjusted using Tukey's HSD for within group comparisons, and Tukey-Kramer for between group comparisons.

## Results

**Figure 1:**
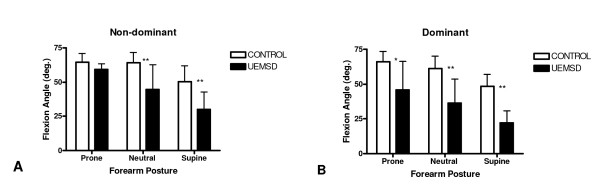
**Mean wrist flexion angle versus forearm posture for the non-dominant (A) and dominant (B) wrists of the UEMSD group (n = 5) and control group (n = 13).** *p < .05, **p < .01. Y-bars = 1 *SD*.

The mean wrist flexion angles for the non-dominant and dominant wrists at each forearm posture for both groups are shown in Figure 1. Dominant wrist flexion in the UEMSD group was significantly restricted compared to the control group at all forearm postures and, in the non-dominant wrist, at the neutral and supine forearm postures. In the dominant wrist, UEMSD group mean flexion angle versus control group mean flexion angle (± 1 standard deviation) was 45.8 (20.6°) versus 66.0 (7.7°), *p *< .05 with the forearm prone; 36.6 (17.2°) versus 61.1 (9.2°), *p *< .01 with the forearm neutral; and 22.2 (8.5°) versus 48.3 (8.7°), *p *< .01 with the forearm supine. In the non-dominant wrist, the UEMSD group's mean flexion angle versus the control group's mean flexion angle was 59.2 (4.1°) versus 64.4 (6.7°), *ns *with the forearm prone; 44.8 (17.9°) versus 64.1 (7.7°), *p *< .01 with the forearm neutral; and 30.0 (12.7°) versus 50.5 (11.4°), *p *< .01 with the forearm supine.

The differences in mean flexion between the three forearm postures within each group are shown in Table 1. Within the control group, mean flexion decreased significantly (*p *< .01) from prone to supine, and from neutral to supine for both wrists. Within the UEMSD group, mean flexion decreased significantly (dominant, *p *< .05; non-dominant, *p *< .01) from the prone to supine forearm posture in both wrists. There were no significant differences in flexion at equivalent forearm postures between the dominant versus non-dominant wrist in either group. None of the UEMSD subjects reported an increase in pain symptoms during the testing procedure.

**Table 1:** Differences in Mean Wrist Flexion between Forearm Postures within Group.

Group	Side	Posture Comparison	Mean Difference	SD (°)	*q*	*df*	Tukey's HSD
Control	Non-Dominant	Prone-Neutral	0.3	8.4	0.13	3,72	ns
		Prone-Supine	13.9	10.0	5.77	3,72	p < .01
		Neutral-Supine	13.6	13.2	5.64	3,72	p < .01
	Dominant	Prone-Neutral	4.9	10.3	2.04	3,72	ns
		Prone-Supine	17.7	11.4	7.33	3,72	p < .01
		Neutral-Supine	12.8	8.1	5.29	3,72	p < .01
UEMSD	Non-dominant	Prone-Neutral	14.4	14.5	2.19	3,24	ns
		Prone-Supine	29.2	11.0	4.45	3,24	p < .01
		Neutral-Supine	14.8	11.8	2.25	3,24	ns
	Dominant	Prone-Neutral	9.2	8.1	1.40	3,24	ns
		Prone-Supine	23.6	16.6	3.60	3,24	p < .05
		Neutral-Supine	14.4	11.3	2.19	3,24	ns

## Discussion

We compared maximum active wrist flexion in UEMSD subjects versus control subjects at three forearm postures. None of the UEMSD subjects reported any increase in pain symptoms during testing, indicating that their wrist flexion was not limited by pain. Mean wrist flexion in the UEMSD group was restricted compared to the control group in both wrists at all forearm postures with the exception of the non-dominant wrist with the forearm prone. These flexion limitations are consistent with previous studies of individuals with UEMSDs [13-17]. However, the most striking result was the decrease in wrist flexion at the supine forearm posture compared to the prone forearm posture in both wrists of both groups.

The control group's mean flexion data for both wrists at the prone and neutral forearm postures was comparable to previous studies of active wrist flexion [24-26]. The control group's flexion decreased at the supine posture compared to the prone or neutral postures in both wrists. Wrist flexion in the UEMSD group also decreased between the prone and supine forearm postures, but to a greater extent than in the control group. Overall, wrist flexion between the prone and supine forearm postures in the UEMSD group decreased 51.5% in the dominant and 49.3% in the non-dominant wrist, and in the control group decreased 26.8% in the dominant and 21.6% in the non-dominant wrist.

We are aware of only one previous study conducted by Hewitt in 1928, who found that mean active wrist flexion decreased approximately 12° between the prone and supine forearm postures in normal subjects [27]. However, in Hewitt's study active wrist flexion was approximately 10° – 15° greater than that shown in previous studies or our study [25,26]. We suspect that this discrepancy may be due to the comparatively young age (mean age < 20 yrs.) and exclusively female gender of the subjects in Hewitt's study, both of which have been associated with increased wrist flexion [28]. Hewitt [27] did not explicitly address the decrease in flexion between the prone and supine forearm posture observed in her study, but suggested that agonist muscle efficiency may be greater with the forearm prone versus supine

Another study on the effects of forearm posture found that maximum active wrist flexion decreased slightly (< 5 degrees) when the forearm was semi-prone (45°) versus fully prone (90°) [26]. This decrease was attributed to possible changes in the articular contact of the carpal bones, or increases in trans-carpal ligament tension [26]. Our results did not show a significant decrease in wrist flexion between the prone and neutral forearm postures in either group. However, the decrease in wrist flexion between the prone and supine forearm postures that is evident in both groups cannot be readily explained by changes in articular contact or trans-carpal ligament tension. The results of a cadaveric study [29] examining wrist tendon excursion demonstrated that the normal wrist is capable of 70 degrees of flexion at the prone, neutral, and supine forearm postures when the proximal wrist motor tendons are severed from their origins. Presumably both cadaveric and live wrists possess equivalent trans-carpal ligament and articular contact characteristics.

Forearm posture may exert an effect on wrist flexion due to biomechanical changes that occur with forearm supination. Several cadaveric wrist studies have shown that excursion of the distal tendon of the extensor carpi ulnaris (ECU) muscle during wrist flexion increases approximately three-fold between the prone and supine forearm postures [29-31]. These results have been attributed to an increase in the length of the distal ECU tendon's moment arm due to dorsal displacement of the distal ECU tendon relative to the carpus during forearm supination [29-31]. A three-fold increase in the length of the distal ECU tendon's moment arm corresponds to a three-fold increase in distal ECU tendon excursion to achieve a given wrist flexion angle. It follows that decreased extensibility of the ECU muscle would resist ECU tendon excursion, limiting wrist flexion to a greater degree with the forearm supine versus prone. Decreased extensibility of the ECU muscle would explain the greater loss of wrist flexion with the forearm supine that we observed in the UEMSD group compared to the control group. This apparent decrease in ECU muscle extensibility may be due to increases in active and/or passive tension.

The presence of ECU muscle tonus and an intact connection to the proximal tendon's origin in live subjects might explain why a supine forearm posture limited wrist flexion in our control subjects, but not in studies of cadaveric wrists [29-31]. Similarly, an increase in ECU muscle activity in the UEMSD group compared to controls might also explain why the decrease in flexion between the prone and supine forearm postures in the UEMSD group was approximately double that in the control group. We are not aware of any studies demonstrating increased wrist extensor muscle activity during wrist flexion in UEMSD subjects, although such increased activity may occur. However, it appears that even maximum contraction of the ECU muscle would be incapable of resisting maximum wrist flexion torque.

A study of normal wrists [32] found that maximum wrist extension torque was less than 60% of maximum flexion torque over the range of flexion angles we measured in our study. Other studies have confirmed the superiority of wrist flexion torque, although to lesser degrees, and all were conducted with the forearm prone [33-35]. Presumably the extension torque capability of the ECU muscle would be enhanced when the forearm is supine due to the increase in its moment arm length [29-31].

We are aware of only one study that examined wrist extensor torque with the forearm supine. Ketchum [36] used measures of wrist extensor muscle masses and tendon excursions from cadavers combined with flexion torque from live subjects to estimate the force generated by each muscle. The tendon excursions were measured with the forearm supine, but the ECU muscle was estimated to contribute less than 30% of the total extensor force [36].

It is also possible that the reduction in wrist flexion with the forearm supine was due to a decrease in wrist flexion torque. However, wrist flexor tendon excursion has not been shown to vary significantly between the prone and supine forearm postures [29,30], suggesting that flexion torque would not decrease. As the ECU muscle is only one of three muscles contributing to wrist extension torque it appears unlikely that even maximum activation of the ECU muscle would be capable of opposing wrist flexion torque. Therefore, we infer that the limited wrist flexion exhibited by the UEMSD group compared to the control group with the forearm supine is due to increased ECU muscle passive tension.

Maximum active wrist flexion in the dominant wrist of the UEMSD group was also below normal with the forearm prone, in agreement with previous studies [13,16,17]. However, we are unable to attribute this flexion restriction to increased ECU muscle passive tension. It follows from the studies of wrist motor tendon excursion previously discussed [29-31] that the required ECU tendon excursion for a given wrist flexion angle is reduced three-fold when the forearm is prone versus supine. Consequently, one would expect the wrist flexion angle to increase three-fold with the forearm prone versus supine if ECU muscle passive tension was the limiting factor. Our results showed that dominant wrist flexion in the UEMSD group increased approximately two-fold between the supine and prone forearm posture, and remained below normal. The fact that wrist flexion increased only two-fold and remained below normal despite a three-fold decrease in the required excursion of the distal ECU tendon indicates that ECU muscle tension is not limiting wrist flexion with the forearm prone.

The limited wrist flexion may have been due to a general impairment in dominant wrist flexor muscle strength in the UEMSD group. A study of subjects with medial and lateral epicondylitis found that peak wrist flexion torque was reduced by 13% in the affected versus the unaffected arm [37]. However, the same study found that peak wrist extension torque was still less than 60% of peak flexion torque in both arms [37]. These results suggest that substantial wrist flexor impairment would be required to limit wrist flexion in conjunction with maximum extensor muscle activation. Instead, we propose that dominant wrist flexion in the UEMSD group with the forearm prone is limited by decreased extensibility of the radial wrist extensor muscles: extensor carpi radialis brevis (ECRB) and extensor carpi radialis longus (ECRL). We infer that this is due to an increase in passive rather than active tension because, as described previously, wrist flexion torque exceeds wrist extension torque over the range of wrist joint angles measured in our study [32].

Our finding of restricted wrist flexion in UEMSD subjects compared to normal controls is consistent with the idea that wrist extensor muscle passive tension is elevated in this group [17]. This interpretation agrees with a previous report of palpable increases in wrist extensor muscle tone with wrist flexion in UEMSD patients [13]. The modulation of maximum active wrist flexion by forearm posture is readily explained by known changes in the length of the distal ECU tendon's moment arm [29-31]. The existence of flexion restrictions in the dominant wrist of the UEMSD group at both the prone and supine forearm postures implicates both the ulnar (ECU) and radial wrist extensor muscles (ECRL and ECRB).

Increased wrist extensor muscle passive tension would affect wrist joint dynamics during flexion. We had initially presumed that, as wrist flexion during computer use is minimal, increased joint and muscle/tendon stress might have occurred during domestic or recreational activities, the symptoms of which were then exacerbated by, and perhaps attributed to computer use. However, the evidence for increased ECU muscle passive tension suggests that increased stress could also occur during radial deviation of the wrist, as the ECU muscle despite its name, functions primarily as an ulnar deviator [30,31].

The apparent increase in extensor muscle passive tension exhibited by the UEMSD group may be related to computer use. Mackinnon and Novak [7] suggest that prolonged abnormal postures may affect muscle tension due to muscle length adaptation. It has been shown that animal skeletal muscle immobilized in a shortened state shortens due to the loss of serial sarcomeres, and the rate of such loss increases when the muscle is chronically activated [38,39]. However, the increase in muscle passive tension that follows immobilization has been attributed to qualitative and quantitative changes in the connective tissue surrounding the muscle, and these changes have been shown to precede sarcomere loss [40]. Conceivably, prolonged wrist extension during computer use could result in muscle length and/or connective tissue changes that increase extensor muscle passive tension.

The cross-sectional design of the current study limits our ability to infer causality, particularly as flexion limitations were also evident in the unaffected wrist of the unilaterally affected UEMSD subjects. However, the results of this study suggest a role for elevated extensor muscle passive tension as a factor in the development of UEMSD symptoms in computer users. Computer usage in the control group was minimal, so we cannot determine from this study whether wrist flexion is similarly affected in asymptomatic computer users. The limitations of this study include a small sample size, and a lack of clear case definitions for the UEMSD subjects. The control group was not age and gender matched to the UEMSD group; however, 6 of the 7 female control subjects were within the age range of the female subjects in the UEMSD group.

## Conclusion

The UEMSD group exhibited reduced active wrist flexion compared to the control group that did not appear to be pain related. A supine versus prone forearm posture reduced wrist flexion in both groups, but the reduction was approximately 100% greater in the UEMSD group. We infer that these results are consistent with increased wrist extensor muscle passive tension, particularly in the extensor carpi ulnaris muscle. The effect of a supine forearm posture on wrist flexion, particularly in highlighting flexion restrictions that were not evident with the forearm prone, suggests that this technique may be a useful addition to the standard wrist ROM testing procedure.

## Competing interests

The author(s) declare that they have no competing interests.

## Authors' contributions

RAB and GBR designed the study. RAB conducted the study and analyzed the data. All authors drafted and revised the manuscript and approved the final manuscript.

## Pre-publication history

The pre-publication history for this paper can be accessed here:


